# Comparative Study of Salivary pH, Buffer Capacity, and Flow in Patients with and without Gastroesophageal Reflux Disease

**DOI:** 10.3390/ijerph19010201

**Published:** 2021-12-25

**Authors:** Farah Bechir, Mariana Pacurar, Adrian Tohati, Simona Maria Bataga

**Affiliations:** 1 Doctoral School of Medicine and Pharmacy, George Emil Palade University of Medicine, Pharmacy, Science, and Technology of Targu Mures, 38 Gh, Marinescu Str., 540142 Targu Mures, Romania; farah.bechir@umfst.ro; 2 Faculty of Dental Medicine, George Emil Palade University of Medicine, Pharmacy, Science, and Technology of Targu Mures, 38 Gh, Marinescu Str., 540142 Targu Mures, Romania; mariana.pacurar@umfst.ro; 3 Faculty of Medicine, George Emil Palade University of Medicine, Pharmacy, Science, and Technology of Targu Mures, 38 Gh, Marinescu Str., 540142 Targu Mures, Romania; simona.bataga@umfst.ro

**Keywords:** GERD, dentistry, saliva quantity, salivary pH, salivary buffer capacity

## Abstract

The oral cavity has specific and individualized characteristics, with pH, saliva flow, buffer capacity, temperature, and microorganisms content influencing oral health. Currently, the prevalence of gastroesophageal reflux disease (GERD) is constantly increasing. The objective of this study was to evaluate and compare the saliva quantity at 5 min, salivary pH, and salivary buffer capacity in patients with and without GERD, necessary for establishing the correct dental treatment plan. A Saliva-Check Buffer (GC) kit was used for the determination of salivary variables. The total number of 80 patients included in the study were divided into a study group and a control group, each containing 40 patients. Saliva quantity at 5 min was lower in patients suffering from GERD. The salivary pH of these patients turned to acid values compared to the salivary pH of controls, where the values were within the normal range. In patients with GERD, the determined salivary buffer capacity was low or very low. The use of the Saliva-Check Buffer (GC) kit is a simple, easy, non-invasive and patient-accepted method, which can also be used in the dentist’s office to assess the saliva buffer capacity and pH, variables that are important for establishing a correct dental treatment plan.

## 1. Introduction

The oral cavity has specific and individualized characteristics, with a wide variety of pH, bacterial load, and temperature variation [1,2]. Temperature and pH of the oral cavity and fluids are two significant factors that affect the electrochemical behavior of restorative dental materials used in a correct dental treatment plan [3,4].

Gastroesophageal reflux disease (GERD) is a current affection, with a prevalence that is constantly increasing [5]. GERD effects are mainly located in the esophagus, but oral cavity disorders are frequently caused by this disease [6,7]. According to the literature, the disease causes an absence of 2.5 h from work, a 23% reduction in efficiency, and a 30% reduction in the normal performance of the individual, and the nocturnal appearance of heartburn can cause sleep disorders; in general, there is a significant reduction in quality of life in patients with GERD [8,9].

Saliva is the major component of the aqueous fluid in the oral cavity, and represents a complex mixture of organic and inorganic secretions of the salivary glands, the fluids and substances arising through the gastroesophageal reflux from the upper respiratory tract, the gingival sulcus, food, and blood-derived compounds [10]. The daily amount of saliva produced by the salivary glands varies from 500 to 2000 mL [11]. Saliva alleviates mastication, swallowing, speaking, and lubricates the oral mucosa, providing an aqueous medium for taste perception [12,13], and performs a vital contribution in the protection of the oral cavity tissues from infections and dental caries [14]. Salivary functions can be systematized in five great categories, with relevance in oral cavity homeostasis, and oral health maintenance: lubrication and protection, buffer activity, preserving the integrity of dental hard tissues, antibacterial action, taste, and digestion [10,15].

Saliva flow, saliva buffer capacity, and saliva content in microorganisms represent very significant factors for oral health [16]. The buffer systems of saliva are liable for retaining suitable acid–base equilibrium [11]. Buffer solutions retain an approximately constant pH even when small amounts of either acid or base are added, or when saliva is diluted, being resistant to changes in the oral pH. The normal pH range for resting saliva is between 6.2–7.6 [10,17]. There are three possible buffer systems found in saliva, namely protein buffer, phosphate buffer, and carbonic acid/bicarbonate buffer (with the most important role) [18,19]. Several studies investigated the correlation between the buffering capacity of saliva and caries activity, concluding that incorporating these parameters in tests during oral examination could predict and detect dental caries risk, and update dental treatment protocols, improving oral health status in medically vulnerable individuals [20,21,22]. Salivary buffers are able to invert the low pH of dental plaque, and permit oral clearance, therefore obviating the enamel demineralization [17]. Through its buffering capacity, saliva also plays a major role in maintaining the teeth integrity (by controlling the demineralization, by the continuous promotion of enamel remineralization, and by providing the main protection against tooth erosion) [23], and of prosthetic restorations (by preventing the apparition of dental alloys corrosion) [24]. The concentration of buffering systems (mainly bicarbonate) grows with the rate of saliva secretion, and the buffering capacity can be ineffective in the case of low flow of unstimulated saliva [11,17].

In GERD, the reduced pH of the oral fluids influences the characteristics, properties, and behavior of dental materials, leading to a decreased life of prosthetic restorations used in the dental treatment of patients suffering from the disease [24,25].

This research represents the initiation of a serial study in which we evaluate the behavior of dental materials considering the results obtained in GERD patients, necessary for establishing the correct dental treatment plan for this category of patients.

The objectives of this study were: (1) to assess the differences between saliva quantity at 5 min, and salivary pH and salivary buffer capacity in patients with and without GERD; and (2) to determine if there is a correlation between the studied salivary parameters. The null hypothesis tested was that GERD would not affect the studied salivary variables.

## 2. Materials and Methods

The study received the approval of the Ethics Commission of Scientific Research within the George Emil Palade University of Medicine, Pharmacy, Science, and Technology from Targu Mures, according to decision no. 807, from 18 March 2020. Also, we obtained the approval of the study following the decision of the Medical Ethics Commission for the Clinical Study of the Drug within SCJU Targu Mures no. Ad. 37243 of 13 December 2019, in accordance with the Helsinki Declaration principles.

The patients included in the study group were selected from the Gastroenterology department of SCJU Targu Mures, and were previously diagnosed with gastroesophageal reflux disease by the department’s specialists. Patients gave their consent after reading the patient information bulletin, and signed the informed consent form for participation in the research. Clinical determinations were performed from March 2020 to March 2021, with a 3-month break caused by the epidemiological context of the COVID-19 pandemic.

Inclusion criteria for the study group patients were represented by male and female patients, at least 18 years of age, diagnosed with GERD (two or more episodes per week), non-smokers or light smokers (less than 10 cigarettes per day), who have agreed to participate in the study, and who have signed the patient’s informed consent. Exclusion criteria were represented by patients under 18 years of age; alarm symptoms, such as dysphagia/odynophagia, anorexia, anemia, unintentional weight loss, or upper gastrointestinal bleeding; patients with malignancies or other serious conditions which alter the general state of health; individuals with post radiation xerostomia or dryness associated with autoimmune disease; patients with salivary gland disorders (e.g., Sjögren’s syndrome, sialolithiasis, sialadenitis); patients undergoing treatment that influences saliva properties (antibiotics, cortisone, anticonvulsants, antiparkinsonian agents, antipsychotics, antidepressants, anxiolytics, antihistamines, antihypertensives, expectorants, decongestants, diuretics, narcotics, monoamine oxidase inhibitors, sedatives, systemic bronchodilators, cardiac antiarrhythmics); Proton Pump Inhibitor (PPI) therapy; pregnancy; heavy smokers; and uncooperative patients who refused to be included in the study (Table 1).

The control group included patients without gastrointestinal pathology who did not follow treatments that influence salivary pH, who agreed to participate in the study, and signed the patient’s informed consent. 

**Figure 1:**
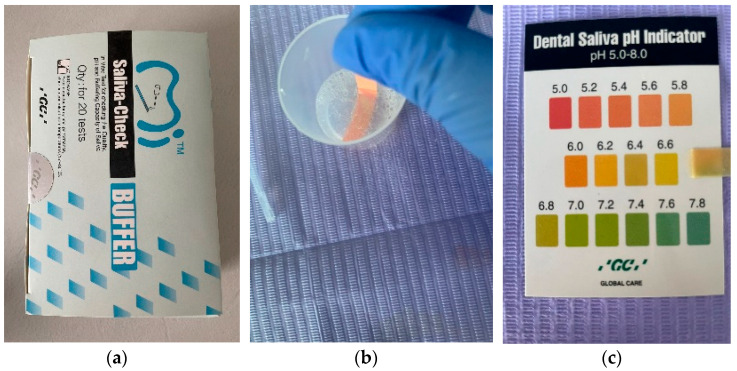
(**a**) GC Saliva Check Buffer kit; (**b**) Placing the pH test strip; (**c**) Obtaining the pH value.

The collection of saliva samples, both stimulated and unstimulated, and the determination of salivary pH and salivary buffer capacity were performed using GC Saliva Check Buffer kits (GC, Tokyo, Japan) (Figure 1a) by a single examiner, in order to avoid calibration errors. The Saliva Check Buffer kit contains 20 in vitro pH test strips, 20 saliva dispensing cups, 20 wax gum pieces for saliva stimulation, 20 saliva dispensing pipettes, 20 buffer test strips, a testing chart for determining the pH and saliva buffer capacity obtained, and instructions for use. The saliva was collected in the morning, after 12 h/overnight fasting, and before eating or drinking any liquids besides water. The patients were instructed not to brush their teeth or use a mouthwash for at least one hour prior to the scheduled appointment time. The patients were instructed to expectorate saliva into the collection cup included in the kit; the resting saliva was visually assessed, and a salivary pH test strip was inserted, which was placed into the sample of resting saliva for 10 s (Figure 1b), and then, the color obtained was compared with the testing chart included in the kit (Figure 1c). PH values above 6.8 correspond to healthy saliva, whereas values between 6.6 and 6 were characterized as moderately acidic, and values below 6 as highly acidic.

**Figure 2:**
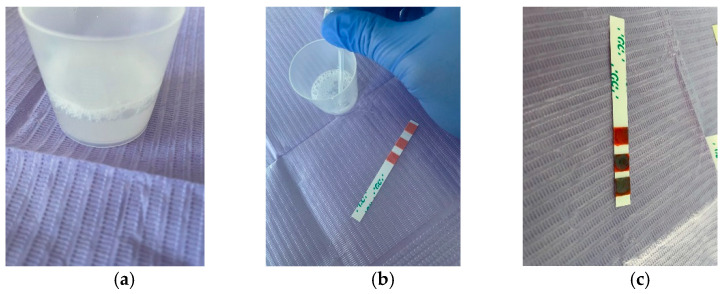
(**a**) Saliva collected in the dispensing cup; (**b**) Use of a pipette to apply saliva to the test strip; (**c**) Buffer test value = 8, low salivary buffer capacity.

Salivary buffer capacity is important because it shows the effectiveness of saliva in neutralizing acids in the oral environment, and is determined using special test strips. Patients were asked to chew the wax gums for 5 min, collecting all the saliva into the collection cup at regular intervals. The quantity of stimulated saliva was measured by checking the gradations on the dispensing cup (Figure 2a). Each salivary buffer test strip is disposable and is individually packaged, as shown in Figure 1b. Using the pipette, saliva was collected from the collection cup (Figure 2b), and three drops were applied to the test strip, one drop to each of the three test pads, which was immediately rotated at 90 degrees to remove excess saliva, therefore preventing the excess saliva from swelling on the test pad and possibly affecting the accuracy of the test result. 

The test changed color immediately, and after an interval of 2 min, the final result could be calculated, summing the values of each color as follows (Figure 2c):

- Green: 4 points;
- Green/Blue: 3 points;
- Blue: 2 points;
- Red/Blue: 1 point;
- Red: 0 points.

The result was interpreted using the scheme in the kit, where each resulting total value corresponds to a degree from “very low” to “normal” salivary buffer capacity, as follows: 0–5: very low; 6–9: low; 10–12: normal.

Statistical analysis was performed in a dedicated software for statistical processing, SPSS24. The differences between the two groups, patients and controls, are established based on specific tests, t-Student for quantitative data, and chi-squared test for qualitative variables. The descriptive statistics of the quantitative variables are highlighted through mean values, whereas for the qualitative variables, the frequency was used. The significance level considered is 0.05. 

## 3. Results

The total 80 patients included in the study were divided into the study group (n = 40) and the control group (n = 40). Table 2 presents the descriptive statistics of the variables for the entire studied population.

The distribution of patients in the entire population is predominantly female, i.e., 48 patients (60%) and 32 men (40%), respectively. On average, the age is 33 years old, and deviates on average from this value by 11 years (SD). The age that divides the entire population into two equal groups is 30 years (median). On average, the salivary pH of the entire population is 6.29, with a standard deviation (SD) of 0.7 units. The median value is 6.4. The average amount of saliva is 7.1 units, with a standard deviation of 1.68 units, and a median value of 7 units. The mean buffer capacity value of the stimulated saliva is 9.25, with a standard deviation of 1.57 units. The median buffer capacity is 10 units.

Table 3 presents the descriptive statistics for the two groups. *p*-values correspond to the t-Student and chi-squared comparison tests between the two groups of patients.

**Figure 3:**
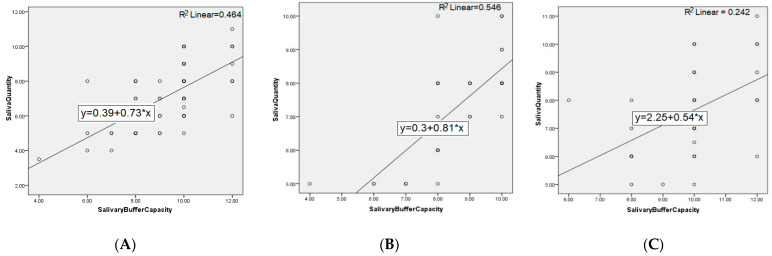
Correlation between salivary flow and buffer capacity for: (**A**) entire population, (**B**) GERD patients, and (**C**) controls.

**Figure 4:**
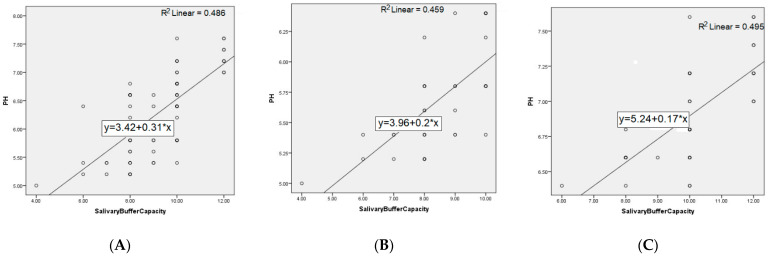
Correlation between pH and salivary buffer capacity for: (**A**) entire population, (**B**) GERD patients, and (**C**) controls.

The distribution of patients by sex in the two groups does not differ significantly (*p*-value = 0.49); respectively, the 40 patients in each group are divided into 18 women (45%) and 22 men (55%) in the GERD group, and 14 women (35%) and 26 men (65%) in the control group. The age of the patients in the two groups did not differ significantly (*p*-value = 0.44). There are statistically significant differences (*p*-value = 0) between the average values of the pH level in the two groups, 5.71 units in patients and 6.88 in controls. The quantity of stimulated saliva at 5 min in the two groups differs significantly (*p*-value = 0.006); respectively, 7.27 units in patients and 7.61 in controls. The buffer capacity of the stimulated saliva in the GERD group is, on average, 8.57, and in the controls is 9.92, statistically different values (*p*-value = 0). The correlations between the studied parameters, both at the level of the entire population and on each group of patients separately, are further represented in Figure 3 and Figure 4.

Distribution of salivary parameters according to normal preset intervals.

For pH, 49 of the entire population fall within the limits of 6.2–7.6 units (61.25%), 9 of which represent GERD patients (22.5%), and all 40 subjects in the control group (100%).

Table 4 shows the distribution of the saliva buffering capacity values in the entire population and each group, respectively.

100% of patients fall into a normally stimulated salivary flow, although the values in GERD patients are significantly lower than those in controls. At the visual inspection of the resting saliva collected in the cup, we observed an increased viscosity, and the presence of residues in patients with GERD, and predominantly watery clear saliva in healthy patients.

We mention that because of the epidemiological context in the COVID-19 pandemic, we could not correlate the oral status (health of the mucosa and hard dental tissues) with the salivary parameters in patients with and without GERD, and were practically limited in our study by the pandemic.

These results suggest that GERD influences patients’ saliva, which may have an impact on the structure of tooth enamel (causing dental erosions), as well as on the properties of dental materials used for restorations (due to the possible apparition of corrosion and tarnish). The null hypothesis tested was rejected.

## 4. Discussion

The present study revealed that GERD has effects on the salivary parameters, namely the unstimulated saliva pH, stimulated salivary flow, and acid buffering capacity of saliva. These findings are in accordance with other studies that found a significantly lower pH in the GERD patients saliva compared with healthy subjects, with an average of 4.9 and 6.5, respectively, in the Caruso et al. [26] study, which concluded that in order to examine a presumptive diagnosis of GERD, we must consider a salivary pH at or below 5. In the oral cavity, the presence of GERD is associated with acidic saliva (pH 4.9) versus the near neutral pH of 6.5 in healthy subjects. A significantly lower mean pH value of 6.65 was found in the GERD group compared to control group (7.23) by Sujatha et al. [27]. Lower salivary flow rate and buffering capacity were noticed in these patients, including significant changes in the hard and soft tissues of the oral cavity.

Salivary pH levels of patients with GERD determined by using the G.C. Saliva Check Buffer kit were statistically significant lower before and after therapy than healthy persons in the Balaban et al. study [28], namely 6.127 before treatment versus 7.08 in controls, and 6.707 after treatment versus 7.08 in controls. Patients had significantly lower values of salivary buffer capacity before and after therapy than healthy persons: 5.057 before treatment versus 10.46 in controls, 8.8 after treatment versus 10.46 in controls, *p* < 0.001. Mihailopol et al. [29] recorded values of salivary pH between 6 and 6.6 for the study group, salivary buffering capacity values ranging from 2 to 6, which are inferior comparing with a normal range of 10–12, as well as a reduced rate of stimulated salivary flow in patients suffering from GERD, concluding that unstimulated saliva presented a lower pH and a lower buffering capacity.

The stimulated salivary flow has an important role for the clearance and cleaning of oral cavity, preventing formation of bacterial biofilm, and preventing dental erosions due to the buffering capacity [29]. The measurement of the buffer capacity of saliva is a diagnostic challenge due to the complex saliva pH regulating systems [30]. The analysis of unstimulated salivary secretion is a precise procedure for investigating the salivary gland secretion, whereas the stimulated saliva is valuable for the assessment of the functional salivary reserve [15]. This is the rationale on which the saliva buffering capacity was determined in our study based on the stimulated saliva, which is known to have a higher concentration of bicarbonate ions and, therefore, a pH that can reach a value of approximately 8 [11].

A correlation by direct proportionality between salivary buffer capacity and stimulated salivary flow has been reported in the Ichim et al. study [31]. Even if several groups of subjects displayed almost the same value of the stimulated salivary flow, different values of salivary buffer capacity were observed, possibly because the salivary buffer capacity also varies in relationship with other parameters, such as salivary pH or DMFT index. However, the study conducted by Correa et al. [32] found that although non-stimulated and stimulated salivary flow, and salivary pH did not show a significant difference between the two groups (GERD and control), salivary buffering capacity was more reduced in individuals with GERD.

The quantity of stimulated saliva at 5 min we found in our study was lower in GERD patients then in healthy individuals, although the mean value was in the normal range of 5–15 mL (1–3 mL/min) [33]. These results are consistent with current data from the literature. Tanabe et al. (2021) [34] analyzed stimulated saliva by chewing sugar-free gums for 3 min in 22 patients with GERD resistant to proton pump inhibitor (PPI) treatment, and 22 patients responding to treatment, and the amount of saliva accumulated was significantly lower in the PPI-resistant group than in the PPI-responding group, with medians of 3.7 (2.2–6.8) and 4.9 (4.0–7.8) mL, respectively (*p* = 0.029). The same protocol for stimulated saliva collection was used by Koeda et al. [35] in their 2021 study, this time on 31 patients diagnosed with non-erosive reflux disease (NERD), and 31 healthy subjects from the control group. The amount of saliva collected was significantly lower in the NERD group than in the control group, with medians of 4.0 mL/3 min (2.0–6.0) and 6.0 (3.9–8), respectively.

The use of saliva tests is expanding the view in clinical diagnosis, disease control, and decision for patient care, and is useful for novel ways for prediction, promoting preventive dentistry, motivating the patient, and eliminating risk factors for oral health problems [36,37,38]. The Saliva-Check Buffer (GC, Tokyo, Japan) kit is easy to handle, and the determinations made with this kit are non-invasive and consented to without prejudice by patients. Through the determinations made with the help of this kit, the dentist has the possibility to establish an optimal treatment plan and an adequate program for the prevention of dental diseases. At the same time, the patient can be educated so that they can have appropriate behavior in performing and maintaining their oral hygiene. The Saliva Check-Buffer method is decidedly the fastest of the three methods used by Kubala et al. [11], while also being more precise than the Dentobuff Strip System. In addition, Maldupa et al. [39] showed that chair-side diagnostic tests are usable for saliva buffer capacity detection in dental offices, and concluded that the GC Saliva Check Buffer has higher accuracy than the CRT Buffer test. However, a study conducted by Kitasako et al. [40] indicates that the colorimetric tests potentially underestimated the buffering capacity of some samples, due to subjectiveness of the operator’s color perception, and the influence of color vision deficiencies, ambient lighting, and operator experience.

Collaboration between physicians and dentists is strongly advocated to prevent or ameliorate possible adverse oral effects from both endogenous and exogenous acids, and to promote adequate saliva production in patients with GERD [41]. Numerous researches present the correlation between the values of salivary pH, the disturbances in salivary quantity, and the GERD symptoms [33,34,35,42,43]. Oral cavity diseases may be developed as a result of changes in the oral fluid characteristics, including pH, which can modify the properties of dental materials [1,44,45,46]. Other researchers have highlighted the correlation between salivary pH and changes in the characteristics of tooth structure and in the restorative dental materials [42,43,47,48,49].

GERD can be appreciated as an important etiopathogenic element in salivary dysfunction, and patients with GERD present a great risk of salivary disturbances [29]. Variations of saliva pH and salivary buffering capacity in patients with GERD can induce structural changes in the composition of the materials from which dental restorations are achieved. Dental restorative materials should present resistance in an acidic environment, a property that should be considered when their selection is realized by dentists [50], because the dental materials should not harm the orofacial tissues [51,52,53]. When properly handled and placed, all dental materials should be biocompatible, to ensure and maintain the patient’s health [52,54,55]. This is the reason why the determination of salivary pH in patients with GERD is very important for our research of compatible dental biomaterials that perform best in the oral conditions of these patients. 

## 5. Conclusions

Within the limitations due to the number of participants in the study, the short timeframe of research, and the pandemic restrictions, we can conclude the following:

- The use of the Saliva-Check Buffer (GC, Tokyo, Japan) kit was a simple, easy, non-invasive and patient-accepted method, and can also be used in the dentist’s office to assess cariogenic risk by testing the quality of pH and buffer capacity of saliva.
- Saliva quantity at 5 min was lower in patients suffering from GERD.
- Salivary pH of patients suffering from GERD turned to acid values, compared to the salivary pH of patients belonging to the control group, without gastrointestinal pathology, where the values were within the normal range.
- In patients with GERD, the determined salivary buffer capacity was low or very low.
- For a correct dental rehabilitation treatment of patients affected by GERD, they should be monitored for a longer duration, and a multidisciplinary approach should be adopted in their treatment.

## Figures and Tables

**Table 1:** Inclusion and exclusion criteria in the study group.

Inclusion Criteria	Exclusion Criteria
GERD positive diagnosis	Pregnancy Patients with malignancies or other serious conditions which alter the general state of health Alarm symptoms Patients with post radiation xerostomia or dryness associated with autoimmune disease Salivary gland disorders Treatment that influences saliva properties PPI therapy
Male and female patients, at least 18 years of age	Minors
Non-smokers/light smokers	Heavy smokers
Patient’s acceptance to participate in the study, with signed informed consent	Uncooperative patients who refused to be included in the study

**Table 2:** Statistical results referring to the saliva quantity at 5 min, salivary pH, and buffering capacity in the entire studied population.

Variable	Mean	Median	SD	Min	Max
Age	33.46	30	11.34	19	63
PH	6.29	6.4	0.7	5	7.6
Quantity at 5 min	7.1	7	1.68	3.5	11
Buffer Capacity	9.25	10	1.57	4	12

**Table 3:** Statistical results referring to the saliva quantity at 5 min, salivary pH, and buffering capacity in the two studied groups.

GERD Patients								Controls						*p*-Value
Variable		Mean	Frequency	Median	SD	Min	Max	Mean	Frequency	Median	SD	Min	Max	
Sex	M		18 (45%)						14 (35%)					0.49
	F		22 (55%)						26 (65%)					
Age		34.45		31.5	10.32	19	63	32.47		30	12.32	19	60	0.44
PH		5.71		5.8	0.41	5	6.4	6.88		6.8	0.34	6.4	7.6	0
Quantity at 5 min		7.27		8	1.53	5	10	7.61		8	1.61	5	11	0.06
Buffer Capacity		8.57		8.5	1.39	4	10	9.92		10	1.47	6	12	0

**Table 4:** Distribution of saliva buffering capacity.

	Saliva Buffering Capacity		
	0–5	6–9	10–12
Entire population	1 (1.3%)	35 (43.75%)	44 (55%)
GERD patients	1 (2.5%)	25 (31.25%)	14 (35%)
Controls	1 (2.5%)	9 (22.5%)	30 (75%)

## Data Availability

Not Applicable.
